# Development of a trigger tool to identify adverse events and no-harm incidents in paediatric oncology: a modified Delphi process using expert knowledge and user experiences

**DOI:** 10.3389/frhs.2025.1731284

**Published:** 2026-01-12

**Authors:** Charlotte Engvall, Margaretha Stenmarker, Ann-Christine Andersson, Axel Ros, Maria Unbeck

**Affiliations:** 1 Department of Biomedical and Clinical Sciences, Linköping University, Linköping, Sweden; 2 Department of Pediatrics, Region Jonkopings lan, Jönköping, Sweden; 3 Department of Pediatrics, Institute of Clinical Sciences, Goteborgs Universitet Sahlgrenska Akademin, Gothenburg, Sweden; 4 Futurum – The Academy for Health and Care, Region Jonkopings lan, Jönköping, Sweden; 5 Jönköping Academy for Improvement of Health and Welfare, School of Health and Welfare, Jonkoping University, Jönköping, Sweden; 6 The Child and Health Care Service, Region Jonkopings lan, Jönköping, Sweden; 7 School of Health and Welfare, Hogskolan Dalarna, Falun, Sweden; 8 Department of Clinical Sciences, Danderyd Hospital, Karolinska Institutet, Stockholm, Sweden

**Keywords:** adverse events, modified Delphi process, no-harm incidents, paediatric oncology, patient safety, retrospective record review, trigger tool

## Abstract

**Background:**

The objective of this study was to develop a Paediatric Oncology Trigger Tool aimed at facilitating the detection of adverse events and no-harm incidents in the patient process from specialised hospital care to home healthcare in paediatric oncology. The development of the trigger tool addresses the need for enhanced safety knowledge in paediatric oncology, particularly as the field has increasingly transitioned from inpatient admissions to day care and home healthcare settings. Existing trigger tools do not fully meet the specific requirements of paediatric oncology, where care is collaboratively provided by patients, parents and healthcare professionals.

**Materials and methods:**

The study employed a multi-step process, including a literature search, a three-phase modified Delphi process, and the practical application of the trigger tool. All six Swedish paediatric oncology centres were represented in the Delphi process. Medical records were reviewed as part of the national multicentre study Patient Safety in Paediatric Oncology, which included participation from four out of six paediatric oncology centres, covering 64% of the population in Sweden. Data were collected from stakeholders representing the patient process from specialised hospital care to home healthcare in paediatric oncology, as well as from reviewers of medical records, and representatives with patient safety and trigger tool methodology expertise. Data were gathered through virtual meetings and web-based surveys, where the triggers were discussed and rated in terms of clinical relevance, comprehensibility and usefulness. Ratings were made using a four-point Likert scale. A dichotomisation process was used to assess consensus, defined as the proportion of respondents giving the same dichotomised rating.

**Result:**

The key outcome was the development of a Paediatric Oncology Trigger Tool. The final tool consisted of 22 triggers with definitions and decision support information, designed to enhance understanding of patient safety in paediatric oncology.

**Conclusions:**

The application of a multi-step development process resulted in a final context-specific trigger tool, the Paediatric Oncology Trigger Tool, addressing unique patient safety needs. The tool can be used in local safety initiatives aiming to improve safety for children with cancer. Additionally, this paper provides a transparent description of a systematic development process.

## Introduction

The World Health Organization defines patient safety as the reduction of risk of unnecessary harm associated with healthcare to an acceptable minimum. An adverse event (AE) is defined as an event that results in harm to the patient, while a no-harm incident is defined as an event that reaches the patient but results in no discernible harm (1). Patient safety extends beyond individual incidents; it encompasses a broader perspective within healthcare systems. This perspective considers patient safety as a discipline within healthcare systems, and as an attribute of the systems that minimises the incidence and impact of AEs and maximises recovery from such events (2). Learning from mistakes and enhancing visibility play crucial roles in patient safety efforts (2, 3).

When identifying healthcare-related complications, often referred to as AEs, a structured retrospective medical record review can serve as a method for extracting data (4, 5). Among the established review methods, the Global Trigger Tool (GTT) developed by the Institute for Healthcare Improvement, is one of the most commonly used (6). The methodology has been found to identify more AEs when compared to other methods (7, 8). The GTT includes a two-step review process. First, two primary reviewers independently review the medical record for the presence of triggers—specific terms or events that could indicate AEs. They then determine whether an AE has occurred, assess its severity, and compare their findings to reach consensus. In the second step, a physician authenticates the reviewerś findings but does not conduct an additional record review (6). The GTT is designed to be used within somatic inpatient care for adults but has also been used without adaptations for evaluating paediatric care (9).

Adaptations from GTT, so-called trigger tools, have been developed for various healthcare settings and focus areas worldwide (4, 10), including paediatric inpatient care (11–16), prehospital emergency care (17) and in- and outpatient oncology care (18). Additionally, a medication trigger tool has been designed to explore adverse drug events in paediatric haematology and oncology patients (19). The method has also been adapted to identify no-harm incidents in adult patients in different settings (20–22) and in paediatric prehospital emergency care (17). Previous work on trigger tool development has highlighted that adaptations are often required to reflect the characteristics of the specific clinical environment (12, 15, 18). In line with this, paediatric oncology presents characteristics that warrant consideration.

Paediatric oncology is a complex and continually evolving clinical field. New treatments continue to emerge, and paediatric oncology care has increasingly shifted from inpatient admissions to day care and home healthcare settings, where care is collaboratively provided by patients, parents and healthcare professionals (23). Remarkable progress has been made, leading to increased survival rates for paediatric oncology patients over the years (24–26). However, children with oncological diseases still face extensive care needs due to their underlying conditions and the treatments they receive, which can result in serious treatment-related complications and patient safety risks (27–31). As paediatric oncology care increasingly extends beyond inpatient care into day care and home healthcare, safety risks arise not only from treatment-related toxicity, which itself is not confined to inpatient care, but also from care transitions, communication failures and complex medication management at home (32). Furthermore, distinctive characteristics of paediatric care, such as pharmacological and physiological differences (33, 34), and childreńs dependence on parents (35–37), create safety challenges that require dedicated consideration with a distinct form in paediatric oncology due to the intensity and complexity of oncological treatment (27). Traditionally, patient safety efforts in paediatric oncology have centred on treatment-related toxicity (30). Knowledge about different types of AEs and no-harm incidents along the broader continuum of care remains limited. Safety knowledge therefore needs to extend beyond treatment-related toxicity to support the development of safety strategies that align with the evolving field (24, 27, 30).

As the GTT was originally developed for adult inpatient somatic care it does not capture the full range of AEs and no-harm incidents occurring in paediatric oncology. Existing adapted trigger tools likewise do not fully address the specific needs of this context, and few are designed to capture no-harm incidents, which are included within the scope of this work. In response to these contextual challenges, the national multicentre study, Patient Safety in Paediatric Oncology (PaSPO), was initiated to increase knowledge about patient safety in paediatric oncology through identifying AEs and no-harm incidents throughout the patient journey from specialised hospital care to home healthcare. As part of PaSPO, the aim was therefore to develop a Paediatric Oncology Trigger Tool (POTT) to facilitate the detection of AEs and no-harm incidents across the continuum of care in paediatric oncology.

## Materials and methods

### Study design

The design of the study to develop a context-adapted trigger tool was based on a literature search, a three-phase modified Delphi process, and experiences captured from a manual record review process. The reporting of the study was guided by the proposed steps of the Accurate Consensus Reporting Document guidelines (ACCORD) (38, 39). The PaSPO multicentre study was led by a research group acting as the steering committee for this study.

### Setting

Paediatric oncology care in Sweden is provided by six geographically dispersed paediatric oncology centres at university hospitals in collaboration with paediatric departments at county hospitals. The patients receive inpatient care, outpatient care, and home healthcare. The latter is sometimes provided by municipality nurses. Four out of six paediatric oncology centres and several county hospitals, whose catchment area corresponds to 64% of the population of Sweden covering both urban and rural areas, participated in this multicentre study. All six paediatric oncology centres were represented in the Delphi process.

### Collation and solicitation of triggers

The development of the POTT was initially inspired by previous work on trigger tools for home healthcare and paediatric hospital care (15, 22). The last author (MU), who was involved in these earlier studies, contributed methodological expertise to ensure consistency and comparability with previously used approaches for trigger tool development. These studies provided a conceptual and methodological foundation for defining and categorising triggers and associated AEs and no-harm incidents.

Building on this foundation, a literature search was conducted in PubMed to identify trigger tools and studies on AEs across a broad range of care contexts, with particular attention to oncology, paediatrics and paediatric oncology across different settings, including both hospital care and home healthcare. The purpose of the search was to identify additional potential triggers, ensure alignment with existing knowledge of AEs within the clinical field, and support the adaptation of the triggers to the specific context of paediatric oncology care. Each potential trigger was linked to associated AEs and no-harm incidents. The findings from the literature search constituted the basis for the first preliminary version of the POTT, which was created in an iterative process by the steering committee. This preliminary version, with triggers, trigger definitions and decision support information, formed the basis of the subsequent Delphi process.

### Prioritisation and refinement of triggers

A modified Delphi process was used to gather the insights of a multidisciplinary group of experts in a structured way (40). Delphi was originally developed in the 1950s and involves a structured iterative process aiming at gaining consensus among experts (41). The modified Delphi method is a common approach in the development of trigger tools in various settings (12, 15, 16, 22, 42). Flexibility exists regarding the design and format of the method (43). In line with several other studies using modified Delphi methods, adjustments were made from the original method regarding the view of anonymity, consensus and the use of virtual meetings in the study (44). The Delphi process included three rounds, including two virtual meetings and a web-based survey.

#### The Delphi panel

Potential expert participants in the Delphi panel were identified by the steering committee through existing networks. The professional experts were purposefully selected with the aim of representing relevant stakeholders and capturing practical and theoretical expertise in patient safety and trigger tool methodology, as well as experiences of paediatric oncology in various geographical and healthcare settings in Sweden. The invitation, with introductory information, was sent to 60 potential participants. One reminder email was sent. Those who responded that they could not participate in the virtual meeting in the first Delphi round were offered the opportunity to contribute in writing. To incorporate the perspectives of patients and parents early in the development of the POTT, four parents with experience in paediatric oncology care were invited to participate in writing in the first Delphi round. The experts contributing to the virtual meeting and/or in writing in the first Delphi round constituted the Delphi panel. To minimise potential bias, none of the members of the steering committee participated in the Delphi panel; however, they acted as facilitators in the meetings.

#### First Delphi round—virtual meeting

Before the virtual meeting, the experts received information, including an overview of the POTT, and information about the study and Delphi methodology. The experts were strategically assigned into four equal-sized groups to achieve representativeness in all groups based on competence and context. The respective group discussions were led by a facilitator from the steering committee; they were recorded, and notes were taken. The triggers were discussed in terms of clinical relevance, comprehensibility and usefulness in the four groups. The experts were also asked to rate the relevance of the triggers and trigger definitions using a four-point Likert scale from “not at all relevant” to “very relevant” and they were given the opportunity to suggest improvements. After the meeting, the experts could add assessments of triggers, other than those discussed in their own group, in writing through ratings and freely worded answers. The parents and those experts who did not participate in the virtual meeting answered in writing in the same way. The results of the ratings, the level of consensus and the free-text comments were analysed. The analysis and recommendations from the Delphi panel were compiled and discussed by the steering committee and revisions of the POTT were made.

#### Second Delphi round—web-based survey

A web-based survey was used, including the full revised POTT. The questions were aimed at capturing the relevance of the triggers and the comprehensibility of the trigger definitions. Ratings were made using a four-point Likert scale. The experts were given the option to answer “no opinion” and to contribute freely worded answers. In addition, the expertś self-reported experiences of paediatric oncology, trigger tool methodology, and patient safety, as well as their experiences of participating in the Delphi process, were rated. The survey was internally pilot-tested by the steering committee for content, layout and wording. The Delphi survey was distributed electronically using the web-based system “esMaker®”. One reminder email was sent. The “esMaker®” system facilitates anonymised feedback from each expert, which was utilised in this study. The results of the ratings and the free-text comments were compiled, analysed and discussed by the steering committee. A refined POTT was produced and used in the record review process.

### Capturing experiences from the use of triggers

In this phase, the medical record reviewers' experiences with the POTT in PaSPO were captured. The reviewers were identified by the steering committee through existing networks. Before the main record-review phase, a pilot test was conducted in which one reviewer examined 29 medical records representing patients diagnosed at one paediatric oncology centre. The purpose of the pilot was to assess the feasibility of the preliminary POTT in a real-world setting. Subsequently, the reviewers and the steering committee convened in a virtual meeting to discuss the clinical relevance, comprehensibility and usefulness of the triggers based on the pilot test results and the revieweŕs gained experience. The reviewers were invited to provide additional improvement suggestions in writing after the meeting. The discussions and written comments were compiled, analysed and discussed by the steering committee, resulting in a refined POTT. In the third step, the revised POTT was used in the main record review process in PaSPO. Medical records representing patients of different ages, diagnoses and types of treatment were reviewed across various healthcare settings, including paediatric oncology centres at university hospitals, paediatric departments at county hospitals and home healthcare. No specific time limit was imposed on the reviewers for completing the assessment of each medical record.In the fourth step, a focus group interview and a web-based survey were conducted to capture the reviewerś experiences. At the time of the survey, most medical records in PaSPO had been reviewed. The survey was internally pilot-tested in the same way as in Delphi round two. The reviewers rated the relevance and usefulness of the triggers, as well as the comprehensibility of each trigger definition and decision support information, using a four-point Likert scale. They also had the opportunity to submit free-text comments for each trigger. The survey was distributed electronically using the web-based system “esMaker®”, with one reminder email sent. The reviewerśexperiences with the POTT are elaborated further in a separate qualitative study (45). The findings from the virtual meeting discussions, the web-based survey, and input from the focus group interview were compiled, analysed and discussed by the steering committee. A refined POTT was then produced for the third Delphi round.

### Final refinement of triggers

#### Third Delphi round—virtual meeting

In the third round, a smaller group of experts from the original Delphi panel was selected through purposeful sampling. The aim was to discuss, refine and anchor the POTT with stakeholders connected to national networks and organisations from the first two rounds. Prior to the virtual meeting, the experts received information including the refined POTT, details about the study and the Delphi methodology. During the virtual meeting, the triggers were discussed in terms of clinical relevance and usefulness. Those unable to attend the virtual meeting were invited to provide assessments of triggers and improvement suggestions through freely worded comments. The discussions and the written comments were compiled, analysed and discussed by the steering committee and revisions of the POTT were made, resulting in the final POTT.

### Data analysis

Data from the web-based surveys were recorded in the survey tool software “esMaker®”. Ratings on Likert scales were converted into numerical values for analysis and presented as median values and min-max. A dichotomisation process was used to assess the consensus of the ratings, merging ratings of “one” and “two” as “low ranking” and “three” and “four” as “high ranking”. Consensus level was defined as the proportion of respondents giving the same dichotomised rating, ranging from 0.5 to 1, indicating either low or high ranking. Triggers with a median relevance rating below three, or those not reaching a consensus level of 0.80, were classified as “triggers to remove”. These could also be classified as “ambiguous triggers” if deemed valuable to retain based on steering committee discussions about clinical relevance in patient safety work. The remaining triggers were classified as “triggers to retain”. Data were analysed using IBM SPSS Statistics (RRID:SCR_016479).

### Ethical approval

The study was conducted in accordance with international research standards and was approved by the Swedish Ethical Review Authority (2020-00116, 2021-03512).

## Result

### Delphi panel

The Delphi panel for this study included 36 professional experts with diverse backgrounds and 3 parent experts, representing relevant healthcare settings, national networks and organisations. Participation in the first Delphi round occurred either through the virtual meeting or by providing written input, with some experts contributing in both ways. The parents contributed through written input only (Table 1).

**Table 1:** Characteristics of the steering committee, the reviewers and the Delphi panel.

Characteristics	Steering committee, *n*	Reviewers, *n*	Delphi panel (Delphi 1–2), *n*	Delphi panel (Delphi 3), *n*
Participants^a^	5	8	39	4
Participation in virtual meeting(s) and writing in Delphi			9	
Participating in virtual meeting in Delphi			18	3
Participating in writing in Delphi			12	1
Experience and knowledge profile^b^				
Patient safety^c^	2	3	12	1
Paediatric Oncology/Paediatrics	2	3	23	3
Parent^d^			3	
Other^e^	1	2	11	
Profession				
Physician	3	2	20	3
Registered nurse^f^	2	4	14	1
Medical student^g^	-	2		
Pharmacist			2	
Work setting				
University hospital^h^	1	2	10	2
County hospital^h^	3	2	13	1
Primary care/Home healthcare			5	
Other^i^	2	2	8	1

^a^ Two participants of the steering committee were also reviewers. Two of the reviewers were also members of the Delphi panel.

^b^ The experts may have more than one expert and knowledge profile.

^c^ A majority of the experts in patient safety had experience and knowledge of trigger tool methodology.

^d^ Parents were not included in Delphi rounds 2 and 3.

^e^ Examples of other experience and knowledge profiles were oncology, pharmacology, psychiatry and intensive care medicine.

^f^ Improvement leaders trained as nurses were included.

^g^ Two of the reviewers were medical students without specified profiles and work settings.

^h^ One of the researchers had two work settings.

^i^ Examples of work settings were university institutions and healthcare administrations.

In the second round, 30 of the 36 invited experts participated. In this round, the experts also reported their self-assessed expertise in key domains. Most reported at least some experience in paediatric oncology (90%), patient safety (97%), and trigger tool methodology (60%). A substantial proportion indicated considerable or extensive experience in these areas (57%, 57% and 30%, respectively). In addition, respondents provided feedback on the Delphi activities. Among those who provided ratings, respondents reported high or very high agreement that the virtual meeting in the first round (53%), the written exercise in the first round (47%), and the web-based survey in the second round (63%) were appropriate ways of contributing to the development of the POTT. Between 23% and 47% selected “no opinion” for these items, which was expected given that not all respondents had participated in every activity. In the third round, four out of seven invited experts participated.

In summary, the panel comprised individuals with varied background and experience, and their feedback reflected engagement with the different Delphi activities.

### Development of evolving POTT versions

**Figure 1:**
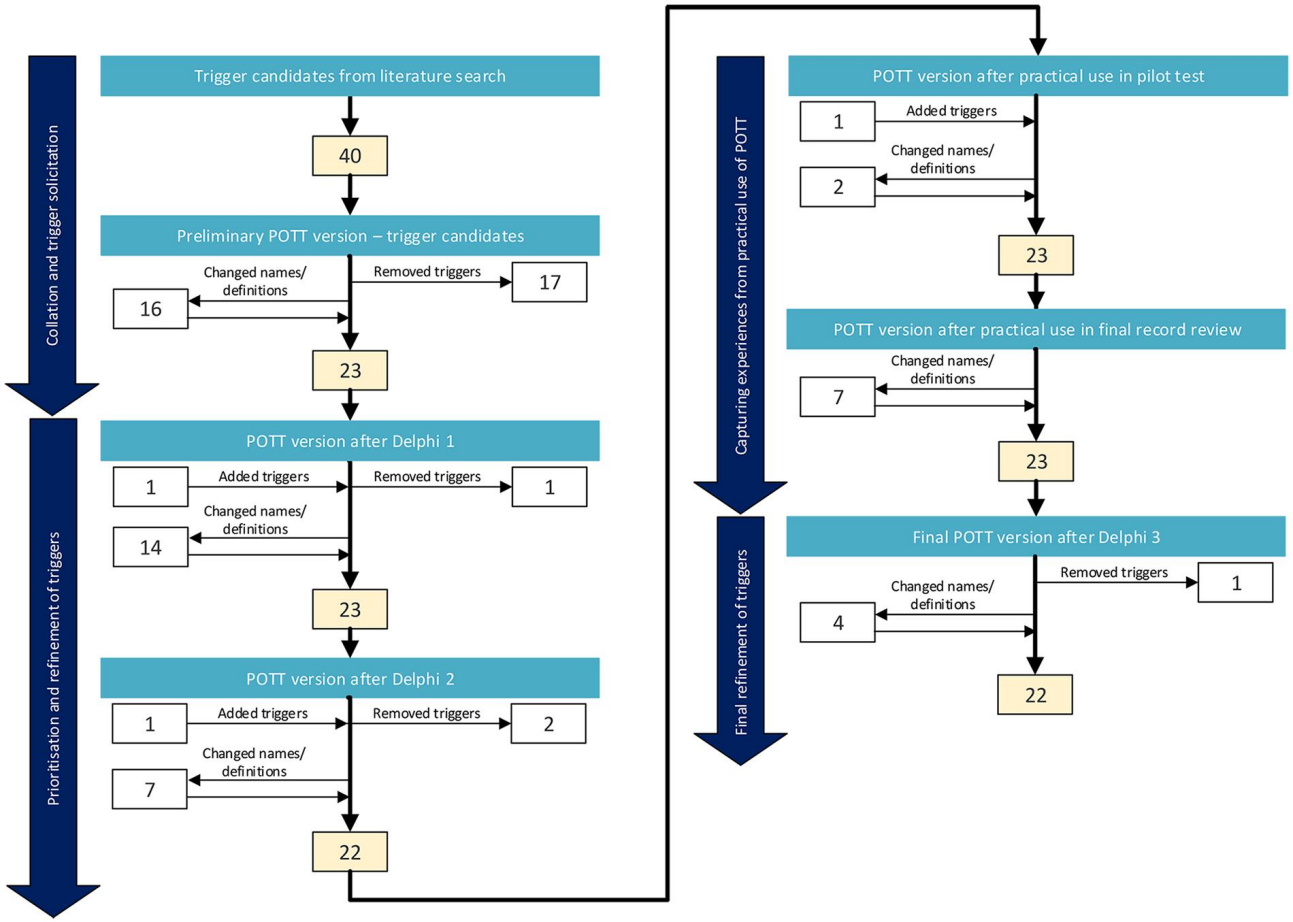
The development of a paediatric oncology trigger tool, POTT.

To complement the tabulated results, the narrative below summarises the main revisions to the POTT across the successive development stages. It outlines how feedback from the Delphi panel and the medical record reviewers guided the refinement of the POTT, while ensuring that decisions were aligned with used principles in trigger tool development. An overview of the results from various steps in the development process is presented in Figure 1.

#### Results of Delphi rounds 1 and 2

Written input was submitted in the first Delphi round, including contributions from parent experts. Their comments addressed issues such as unplanned contact with healthcare providers, the burden placed on parents in coordinating care, and appreciation for triggers that reflected aspects of continuity and communication. They also emphasised the importance of recognising psychological distress, illustrated through comments on the psychological side-effects of high-dose corticosteroid treatment and examples of withdrawal symptoms.

In the first round, the median values regarding the relevance of the triggers ranged from 2.5 to 4. The achieved consensus level ranged from 0.5 to 1.0 (Table 2). Triggers such as “distended urinary bladder”, “fall”, “invasive procedure”, “drug that requires follow-up” and “drug management” were classified as ambiguous triggers included in the subsequent version of the POTT. The trigger “unplanned contact with physician and registered nurse”, was classified as a trigger to remove. Additionally, the trigger “patient treated off-site” was added (Table 2 and Figure 1).

**Table 2:** Result of the Delphi rounds

Preliminary triggers	Delphi Round 1						Delphi Round 2							Delphi round 3	
	Relevance of triggers		Relevance of trigger definitions		Changed trigger names or definitions after Delphi round 1	Result of Delphi round 1	Relevance of triggers		Comprehensibility of trigger definitions		Changed trigger names or definitions after Delhi round 2	Result of Delphi round 2	Added after pilot test after Delphi round 2	Result of Delphi round 3	Changed trigger names or definitions after Delhi round 3
	Median (min–max)	Consensus	Median (min–max)	Consensus			Median (min–max)	Consensus	Median (min–max)	Consensus					
General module															
Cardiac arrest and deterioration in vital functions	4 (3–4)	1.00	4 (2–4)	0.94	Yes	Retained	4 (3–4)	1.00	4 (2–4)	0.97	Yes	Retained		Retained	Yes
Neurological impairment	4 (2–4)	0.90	4 (3–4)	1.00	No	Retained	4 (3–4)	1.00	3 (1–4)	0.75	No	Retained		Retained	No
Blood vessel, skin and tissue impairment	4 (4–4)	1.00	4 (3–4)	1.00	Yes	Retained	4 (3–4)	1.00	3.5 (1–4)	0.82	No	Retained		Retained	Yes
Renal impairment	4 (3–4)	1.00	3 (2–4)	0.85	Yes	Retained	4 (3–4)	1.00	4 (3–4)	1.00	No	Retained		Retained	Yes
Thrombosis and embolus	4 (4–4)	1.00	4 (2–4)	0.93	No	Retained	4 (2–4)	0.97	4 (3–4)	1.00	No	Retained		Retained	No
Healthcare–associated infection	4 (4–4)	1.00	4 (3–4)	1.00	Yes	Retained	4 (2–4)	0.97	3.5 (2–4)	0.89	Yes	Retained		Retained	No
Gastrointestinal impairment	4 (3–4)	1.00	3 (3–4)	1.00	No	Retained	4 (2–4)	0.96	3 (2–4)	0.81	No	Retained		Retained	No
Impairment of oral health	4 (3–4)	1.00	4 (3–4)	1.00	No	Retained	4 (2–4)	0.96	4 (3–4)	1.00	Yes	Retained		Retained	No
Distended urinary bladder	3 (2–4)	0.65	4 (3–4)	1.00	Yes	Ambiguous	4 (2–4)	0.93	4 (3–4)	1.00	No	Retained		Retained	No
Weight loss	4 (3–4)	1.00	4 (1–4)	0.94	Yes	Retained	4 (2–4)	0.93	4 (2–4)	0.96	No	Retained		Retained	No
Fall	2.5 (1–4)	0.50	3 (1–4)	0.81	No	Ambiguous	3 (2–4)	0.59	4 (2–4)	0.93	No	Ambiguous		Removed	
Pain	4 (4–4)	1.00	3 (3–4)	1.00	Yes	Retained	4 (4–4)	1.00	3 (2–4)	0.89	Yes	Retained		Retained	No
Psychological impairment	4 (3–4)	1.00	3 (2–4)	0.88	Yes	Retained	4 (2–4)	0.96	3 (2–4)	0.96	Yes	Retained		Retained	Yes
Invasive procedure	2.5 (2–4)	0.50	4 (2–4)	0.78	Yes	Ambiguous	4 (2–4)	0.93	4 (2–4)	0.89	No	Retained		Retained	No
Deviating course in the use of medical device	3 (2–4)	0.90	3 (2–4)	0.70	No	Retained	4 (2–4)	0.96	3 (1–4)	0.71	Yes	Retained		Retained	No
Mistake, complaint and incident	4 (4–4)	1.00	4 (2–4)	0.90	Yes	Retained	4 (3–4)	1.00	4 (3–4)	1.00	Yes	Retained		Retained	No
Other	4 (2–4)	0.88	3.5 (2–4)	0.88	Yes	Retained	4 (2–4)	0.93	3 (1–4)	0.79	No	Retained		Retained	No
Transfusion												Added		Retained	No
Endocrine impairment													Added	Retained	No
Medication module															
Adverse drug event/Adverse drug reaction	4 (3–4)	1.00	3 (2–4)	0.71	Yes	Retained	4 (3–4)	1.00	4 (2–4)	0.87	No	Retained		Retained	No
Drug that requires follow–up	4 (2–4)	0.76	2 (1–4)	0.59	No	Ambiguous	4 (1–4)	0.69	4 (2–4)	0.88	No	Removed			
Drug management	3 (2–4)	0.56	3.5 (1–4)	0.75	Yes	Ambiguous	4 (2–4)	0.97	4 (2–4)	0.87	No	Retained		Retained	No
Continuity and transition module															
Unplanned change in care–providing unit, admission and outpatient visit	4 (3–4)	1.00	3 (2–4)	0.67	No	Retained	4 (1–4)	0.85	4 (1–4)	0.82	No	Retained		Retained	No
Unplanned contact with physician and registered nurse	3 (1–4)	0.75	3 (2–4)	0.87		Removed									
Insufficient planning, coordination, communication and information	4 (4–4)	1.00	3.5 (3–4)	1.00	Yes	Retained	4 (2–4)	0.83	4 (2–4)	0.93	No	Retained		Retained	No
Patient treated off–site						Added	4 (1–4)	0.97	4 (2–4)	0.90	No	Removed			

In the second round, the median relevance values for all triggers were 4. The achieved consensus level ranged from 0.59 to 1.0 (Table 2). The trigger “fall” was classified as an ambiguous trigger again and remained in subsequent versions of the POTT. The trigger “drug that requires follow-up” was classified as a trigger to remove. Based on free-text comments and verbal discussions, the trigger “patient treated off-site”, which had been added in the first round, was identified as a trigger to remove and the trigger “transfusion” was included (Table 2 and Figure 1).

#### Results of capturing the experiences from use of the POTT

Additional insights that complemented the Delphi findings were obtained from the reviewers during the subsequent use of the POTT in the PaSPO study. Evaluation of the experiences and discussions after the pilot test led to clarifications of several trigger definitions and decision support information. An additional trigger, “endocrine impairment”, was added (Table 2 and Figure 1).

All eight reviewers participated in the web-based survey, which was conducted after the majority of the medical record reviews had been completed. The median values regarding the relevance of the triggers ranged from 3 to 4. The achieved consensus level ranged from 0.63 to 1 (Table 3).The trigger “fall” was again classified as ambiguous and included in the subsequent version of the POTT.

**Table 3:** Result of the reviewers' survey

Modules and triggers	Relevance of triggers		Usefulness of triggers		Comprehensibility of trigger definitions		Comprehensibility of decision support		Result of reviewers’ survey	Changed trigger names or definitions
	Median (min–max)	Consensus	Median (min–max)	Consensus	Median (min–max)	Consensus	Median (min–max)	Consensus		
General Module										
Deterioration in vital functions	4 (3–4)	1.00	3 (3–4)	1.00	4 (3–4)	1.00	4 (3–4)	1.00	Retained	No
Neurological impairment	4 (4–4)	1.00	4 (3–4)	1.00	4 (3–4)	1.00	4 (4–4)	1.00	Retained	No
Blood vessel, skin or tissue impairment	4 (3–4)	1.00	4 (3–4)	1.00	4 (2–4)	0.75	4 (3–4)	1.00	Retained	Yes
Renal impairment	3.5 (3–4)	1.00	3 (2–4)	0.88	4 (3–4)	1.00	4 (3–4)	1.00	Retained	No
Thrombosis or embolus	4 (3–4)	1.00	4 (2–4)	0.75	4 (4–4)	1.00	4 (4–4)	1.00	Retained	Yes
Healthcare–associated infection	4 (2–4)	0.88	4 (2–4)	0.88	4 (3–4)	1.00	4 (2–4)	0.88	Retained	Yes
Gastrointestinal impairment	4 (4–4)	1.00	4 (3–4)	1.00	4 (3–4)	1.00	3 (3–4)	1.00	Retained	No
Impairment of oral health	4 (4–4)	1.00	4 (4–4)	1.00	4 (4–4)	1.00	4 (3–4)	1.00	Retained	No
Distended urinary bladder	3.5 (2–4)	0.88	3 (2–4)	0.88	4 (3–4)	1.00	3.5 (3–4)	1.00	Retained	No
Weight loss	4 (3–4)	1.00	4 (3–4)	1.00	4 (2–4)	0.88	3.5 (3–4)	1.00	Retained	No
Fall	3 (2–4)	0.63	3 (2–4)	0.75	4 (3–4)	1.00	4 (3–4)	1.00	Ambiguous	No
Pain	4 (4–4)	1.00	4 (3–4)	1.00	4 (4–4)	1.00	4 (3–4)	1.00	Retained	No
Psychological impairment	4 (2–4)	0.88	4 (2–4)	0.88	4 (3–4)	1.00	4 (3–4)	1.00	Retained	No
Unplanned invasive procedure or deviating course in invasive procedure	4 (3–4)	1.00	3.5 (2–4)	0.88	4 (2–4)	0.88	4 (3–4)	1.00	Retained	No
Deviating course in the use of medical device	4 (3–4)	1.00	3.5 (3–4)	1.00	4 (2–4)	0.88	4 (3–4)	1.00	Retained	No
Mistake, complaint and incident	4 (4–4)	1.00	4 (3–4)	1.00	4 (4–4)	1.00	4 (3–4)	1.00	Retained	No
Transfusion	4 (3–4)	1.00	4 (2–4)	0.63	4 (4–4)	1.00	4 (3–4)	1.00	Retained	Yes
Endocrine impairment	4 (3–4)	1.00	3.5 (3–4)	1.00	4 (3–4)	1.00	4 (3–4)	1.00	Retained	No
Other	4 (2–4)	0.88	4 (2–4)	0.63	4 (3–4)	1.00	4 (3–4)	1.00	Retained	Yes
Medication module										
Adverse drug event/Adverse drug reaction	4 (4–4)	1.00	4 (2–4)	0.88	4 (4–4)	1.00	4 (3–4)	1.00	Retained	No
Deviating course in drug management	4 (4–4)	1.00	4 (3–4)	1.00	4 (2–4)	0.88	4 (3–4)	1.00	Retained	No
Continuity and transition module										
Unplanned change in care–providing unit	3.5 (2–4)	0.88	2.5 (2–4)	0.50	4 (2–4)	0.88	4 (2–4)	0.88	Retained	Yes
Insufficient planning, coordination, communication and information	4 (2–4)	0.88	3.5 (2–4)	0.88	4 (1–4)	0.88	4 (3–4)	1.00	Retained	Yes

#### Results of delphi round 3

In the third Delphi round, the final refinements were agreed upon, resulting in the final version of the POTT. The trigger “fall”, previously classified as ambiguous, was removed. No triggers were added (Table 2 and Figure 1).

### The final trigger set for the POTT

The final POTT consisted of 22 triggers, along with definitions and decision support information (Table 4). Substantial changes to the wording of trigger names and definitions were made until Delphi round 2. After practical use of the POTT had begun, only minor changes to the wording were made. The changes were based on the steering committeés analysis of group discussions and ratings regarding the usefulness, relevance and comprehensibility of the triggers, trigger definitions and decision support information (Tables 2, 3).

**Table 4:** The final trigger set for the paediatric oncology trigger tool.

Final trigger set
General Module
Deterioration in vital functions
Neurological impairment
Blood vessel, skin or tissue impairment
Renal impairment
Thrombosis or embolus
Infection
Gastrointestinal impairment
Impairment of oral health
Distended urinary bladder
Weight loss
Pain
Psychological impairment
Unplanned invasive procedure or deviating course in invasive procedure
Deviating course in the use of medical device
Mistake, complaint or incident
Transfusion
Endocrine impairment
Other
Medication module
Adverse drug event/Adverse drug reaction
Deviating course in drug management
Continuity and transition module
Unplanned change in care-providing unit
Insufficient planning, coordination, communication or information

## Discussion

The POTT was developed using a rigorous and systematic multi-step approach, including literature reviews, a three-phase modified Delphi process, and insights gained from practical use. This process resulted in a tool with 22 triggers, corresponding definitions and decision support information designed to facilitate the detection of AEs and no-harm incidents in the patient process and enhance understanding of patient safety in the complex field of paediatric oncology.

The need for a context-specific trigger tool aligns with existing literature (9, 12, 15, 18). Our decision to develop a specific POTT reflects the unique challenges in paediatric oncology care and is strengthened by the fact that a significant burden of AEs has been identified using a context-specific trigger tool in adult oncology care (46).

It is strongly recommended to include paediatric high-risk populations and high-alert medications in medication safety research, consistent with our study (34). Adapting adult-focused trigger tools for paediatric use without modifications is not advisable, as noted over a decade ago (9). While several research groups have developed trigger tools for paediatric inpatient setting (11–16), our study is, to the best of our knowledge, the first to create a trigger tool for a medical record review in paediatric oncology. Our tool is designed for use across the entire care continuum, not just inpatient care; this is necessary to reflect the shift towards day care and home healthcare settings in paediatric oncology (23, 47). This comprehensive approach aligns with the development of a trigger tool for adults with oncological diseases and the call for research including smaller hospitals, as existing evidence primarily comes from tertiary university medical centres (18, 34).

The final version of POTT reflects the contextual requirements of paediatric oncology through its triggers, definitions and, in particular, its decision support information. Throughout the development process, the content was informed by considerations important to paediatric oncology. These contextual influences are especially evident in the decision support descriptions, where examples and guidance were shaped to capture AEs and no-harm incidents relevant across inpatient, day care and home healthcare settings in paediatric oncology. Our development process is distinguished by its robustness and systematic nature. We describe this process transparently, following recently published guidelines (38). By adhering to the ACCORD guidelines, we provide insights for others developing trigger tools. Previous descriptions of paediatric trigger tool development have varied in detail. Some authors mention modifying existing tools without explaining how or why (48–51). Others describe adaptations but not the rationale (14, 52). Some mention using a modified Delphi method but lack transparency about the process (11, 53, 54). These inconsistencies might detract from the value of the developed tools (44). Our development process, and the transparent description of it, builds on the robust methodologies used in developing the Global Assessment of the Pediatric Patient Safety Tool (13), the Pediatric All Cause Harm Measurement Tool (12), the Canadian Paediatric Trigger Tool (16) and the Swedish Paediatric Trigger Tool (15), and benefits from recently published guidelines (38, 39).

The initial literature search grounded our preliminary POTT used in the Delphi process in existing research. We searched not only for trigger tools, but also for AEs of relevance for paediatric oncology. This work, combined with the clinical experience of the steering committee, ensured the tooĺs relevance and is consistent with the preliminary trigger tool remaining relatively stable through the Delphi rounds. We did not conduct a *systematic* literature search, as relevant compilations were already available from previous studies by the last author (15, 22).

The relative stability of the preliminary trigger tool across the Delphi rounds appears to reflect the strong foundation provided by the initial literature search and the high level of agreement among the Delphi experts. Consequently, discussions mainly focused on clarifying definitions and ensuring clinical applicability rather than questioning the inclusion or relevance of the triggers. Discrepancies did arise for triggers requiring contextual interpretation across care settings, such as “fall” and “drug that requires follow-up” where relevance varied between inpatient, outpatient and home healthcare settings.

The Delphi approach is a robust methodology for achieving consensus among experts with diverse skills. The Delphi methodology has been adapted in various forms, leading to debates about certain principles, such as expert selection, anonymity, and the number of rounds (43, 44). We describe our Delphi process thoroughly, in alignment with reporting guidelines (38). This is often lacking in other studies, which might hinder the interpretation of the results (44). Similar to other developers of trigger tools (12, 15, 16, 42), we found the Delphi method useful.

There is no precise guidance on the size or definition of an expert panel (43, 44), although 20–30 experts in a panel are common (38). Heterogeneity in a panel may lead to better performance than homogeneity, and all relevant stakeholders should be represented (44). Our number of experts and their diverse backgrounds made it possible to obtain a wide range of perspectives relevant to the development of the POTT, while still allowing the practical implementation of the Delphi rounds. The steering committee selected the potential experts starting from the criteria that the experts would represent relevant stakeholders and possess relevant expertise. The steering committeés strong connections within relevant stakeholder organisations ensured good knowledge of potential experts. The expertś self-assessment of their experience in paediatric oncology, trigger tool methodology and patient safety indicates that the Delphi panel consisted of experienced experts. Only Swedish experts were included, which can be seen as a limitation. However, paediatric oncology is characterised by international collaborations regarding research, treatment regimens, working methods and professional training, suggesting that generalisation of the POTT from an international perspective and context is possible. This suggestion is further strengthened by the fact that the literature search that formed the basis for the tool included literature from various countries.

Depending on the studýs objective, including patients or patient representatives in the expert panel should be considered (44). Patients and parents are integral to paediatric oncology care, especially with the shift towards home-based care (3, 23, 55). To ensure a comprehensive perspective, parents were invited to participate in writing in the first Delphi round. Their perspectives complemented those of the professional experts by underscoring aspects of safety that may be more apparent to parents than to healthcare professionals, including psychological distress, symptoms associated with medication withdrawal and safety risks arising from gaps in care coordination. However, they were not included in the virtual meeting to avoid inhibiting open discussion of patient safety risks. This decision means some perspectives may be missing. In hindsight, patients and parents would have been involved to a greater extent, reflecting increased readiness and openness to fully involve patient representatives in research (56). Increased patient and parent involvement could further strengthen the understanding of patient safety risks and needs in paediatric oncology, particularly in home and transitional care context.

There is no precise guidance in the literature on the number of Delphi rounds. Ending the process too early risks obtaining invalid or meaningless results (43, 44). We used a model where the same experts, except for the parents, were invited to the first two rounds. The medical record reviewerś feedback about the POTT weighed heavily in the development process, therefore, the third Delphi round was primarily aimed at discussing, refining and anchoring the POTT with stakeholders connected to national networks and organisations. Accordingly, a purposefully selected smaller number of experts from the first rounds, representing these stakeholders, were invited to the final round. The thoughtful selection of invited experts ensured that the final round's purpose was achieved, although the low number of experts can be seen as a limitation. The smaller number of experts in the third round also mitigated the risk of decreased engagement, which can occur if the Delphi process is overly complex or time-consuming (43, 44). Ultimately, each expert was provided with the final version of the POTT.

There is no precise guidance in the literature on how to assess, manage and relate to consensus. A lack of clarity regarding consensus can impair the value of any study (43, 44, 57). Our transparency in assessing and managing how the level of consensus informed the trigger tool development is a strength. The level of consensus reached was not entirely decisive for whether triggers were retained or removed. Ultimately, these decisions were made by the steering committee, which might be perceived as a limitation. However, this modification of the original Delphi method is common and can improve the validity of the developed tool (43, 58). The steering committeés decisions were guided by clinical relevance, patient safety considerations and methodological coherence. The committee reviewed quantitative results together with qualitative comments, and professional judgement was applied to ensure that the final set of triggers was grounded in the study findings, informed by previous research, and supported by collective expertise within paediatric oncology and patient safety. The role of the steering committee also informed our decision to only give feedback on the latest version of the POTT before round two, not including the expert group´s and individualś ratings, which might be seen as a limitation. However, feedback was given in the last round.

The combination of virtual meetings and web-based surveys allowed us to introduce the study to the experts and helped them become familiar with it, which probably increased their commitment, participation and knowledge regarding the study (43, 44). Preservation of the expertś anonymity is a basic requirement in the original Delphi method, aiming to reduce the risk of excessively dominant individuals influencing the result, but adding meetings is a common modification (44). The facilitators were aware of the risk of a few experts dominating the discussions at the expense of others and actively counteracted such a situation by ensuring that everyone was involved in the group discussions. The feedback on the Delphi activities did not indicate any substantial concerns regarding the virtual meeting format.

Despite the technological development enabling virtual meetings and their explosive increase, there is a lack of scientific guidance on how virtual consensus meetings are best conducted (59). The use of a digital platform made it possible for busy and geographically dispersed experts to participate in the same meeting. The lack of scientific guidance was compensated by the steering committeés extensive experience in conducting virtual meetings in various settings.

A Delphi process alone may be insufficient to ensure the validity and reliability of the developed tool and may need to be supplemented with pilot tests or focus groups (43). In this study, involving actual users in the tool's development yielded invaluable insights into their priorities, thereby ensuring that the tool remains responsive to their needs, while relevant information and guidance was offered during the review process. The reviewerś experiences of participating in the development and use of the POTT are further elaborated in a complementary qualitative study (45), which provides a broader perspective on the development process described here.

### Conclusion

#### Implications for practice

This study provides a novel POTT, complete with triggers, definitions, and decision support information. We anticipate that this context-adapted patient safety tool can be utilised in local patient safety initiatives to detect AEs and no-harm incidents in paediatric oncology. Its use in practice may be supported by local strategies such as opportunities for reflection within existing patient safety structures. By gaining new insights into risk areas for children with cancer, the groundwork can be laid for developing safety strategies aimed at enhancing patient safety in paediatric oncology, supported by mechanisms that allow findings to inform both ongoing and emerging safety improvement work, which represent important considerations for the tooĺs use in clinical practice.

Additionally, this paper provides a transparent description of a systematic development process and offers insights for further research.

The paediatric oncology settings in parts of the world where treatment is given according to strict international treatment protocols have much in common. Therefore, we anticipate that the POTT can be utilised internationally, even though the tool was developed in Sweden.

#### Further research

In subsequent publications, we will present the performance and the validation process of the POTT, along with the identified AEs and no-harm incidents and their clinical implications. These forthcoming publications align with the aim of the multicentre study PaSPO, of which this study forms a part.

The interest in developing and using semi- or fully automated AE detection methods is increasing, since less time and personnel resources are required and real-time feedback as a basis for rapid interventions can take place (60). We anticipate that the POTT could be the basis for research and development of automated methods for the detection of AEs in a collaboration between clinicians, patient safety experts and experts in natural language processing.

## Data Availability

The datasets generated and analysed during the current study are available from the corresponding author upon reasonable request.
