# Correction: Development of a trigger tool to identify adverse events and no-harm incidents in paediatric oncology: a modified Delphi process using expert knowledge and user experiences

**DOI:** 10.3389/frhs.2026.1874078

**Published:** 2026-06-08

**Authors:** Charlotte Engvall, Margaretha Stenmarker, Ann-Christine Andersson, Axel Ros, Maria Unbeck

**Affiliations:** 1 Department of Biomedical and Clinical Sciences, Linköping University, Linköping, Sweden; 2 Department of Pediatrics, Region Jonkopings lan, Jönköping, Sweden; 3 Department of Pediatrics, Institute of Clinical Sciences, Sahlgrenska Akademin, Goteborgs Universitet, Gothenburg, Sweden; 4 Futurum Akademin for Halsa Och Vard, Jönköping, Sweden; 5 Jönköping Academy for Improvement of Health and Welfare, School of Health and Welfare, Jonkoping University, Jönköping, Sweden; 6 The Child and Health Care Service, Region Jonkopings lan, Jönköping, Sweden; 7 School of Health and Welfare, Hogskolan Dalarna, Falun, Sweden; 8 Department of Clinical Sciences, Danderyd Hospital, Karolinska Institutet, Stockholm, Sweden

**Keywords:** adverse events, modified Delphi process, no-harm incidents, paediatric oncology, patient safety, retrospective record review, trigger tool

**Error in figure/table**

There was a mistake in table 2 as published. In the row “Insufficient planning, coordination, communication and information,” under Delphi Round 1, relevance of triggers, the min–max entry was incorrectly presented as “1–4”. The correct entry is “4–4”. The corrected table 2 appears below.

TABLE 2 Result of the Delphi rounds.

**Table 2:** Result of the Delphi rounds

Preliminary triggers	Delphi Round 1						Delphi Round 2							Delphi round 3	
	Relevance of triggers		Relevance of trigger definitions		Changed trigger names or definitions after Delphi round 1	Result of Delphi round 1	Relevance of triggers		Comprehensibility of trigger definitions		Changed trigger names or definitions after Delhi round 2	Result of Delphi round 2	Added after pilot test after Delphi round 2	Result of Delphi round 3	Changed trigger names or definitions after Delhi round 3
	Median (min–max)	Consensus	Median (min–max)	Consensus			Median (min–max)	Consensus	Median (min–max)	Consensus					
General module															
Cardiac arrest and deterioration in vital functions	4 (3–4)	1.00	4 (2–4)	0.94	Yes	Retained	4 (3–4)	1.00	4 (2–4)	0.97	Yes	Retained		Retained	Yes
Neurological impairment	4 (2–4)	0.90	4 (3–4)	1.00	No	Retained	4 (3–4)	1.00	3 (1–4)	0.75	No	Retained		Retained	No
Blood vessel, skin and tissue impairment	4 (4–4)	1.00	4 (3–4)	1.00	Yes	Retained	4 (3–4)	1.00	3.5 (1–4)	0.82	No	Retained		Retained	Yes
Renal impairment	4 (3–4)	1.00	3 (2–4)	0.85	Yes	Retained	4 (3–4)	1.00	4 (3–4)	1.00	No	Retained		Retained	Yes
Thrombosis and embolus	4 (4–4)	1.00	4 (2–4)	0.93	No	Retained	4 (2–4)	0.97	4 (3–4)	1.00	No	Retained		Retained	No
Healthcare–associated infection	4 (4–4)	1.00	4 (3–4)	1.00	Yes	Retained	4 (2–4)	0.97	3.5 (2–4)	0.89	Yes	Retained		Retained	No
Gastrointestinal impairment	4 (3–4)	1.00	3 (3–4)	1.00	No	Retained	4 (2–4)	0.96	3 (2–4)	0.81	No	Retained		Retained	No
Impairment of oral health	4 (3–4)	1.00	4 (3–4)	1.00	No	Retained	4 (2–4)	0.96	4 (3–4)	1.00	Yes	Retained		Retained	No
Distended urinary bladder	3 (2–4)	0.65	4 (3–4)	1.00	Yes	Ambiguous	4 (2–4)	0.93	4 (3–4)	1.00	No	Retained		Retained	No
Weight loss	4 (3–4)	1.00	4 (1–4)	0.94	Yes	Retained	4 (2–4)	0.93	4 (2–4)	0.96	No	Retained		Retained	No
Fall	2.5 (1–4)	0.50	3 (1–4)	0.81	No	Ambiguous	3 (2–4)	0.59	4 (2–4)	0.93	No	Ambiguous		Removed	
Pain	4 (4–4)	1.00	3 (3–4)	1.00	Yes	Retained	4 (4–4)	1.00	3 (2–4)	0.89	Yes	Retained		Retained	No
Psychological impairment	4 (3–4)	1.00	3 (2–4)	0.88	Yes	Retained	4 (2–4)	0.96	3 (2–4)	0.96	Yes	Retained		Retained	Yes
Invasive procedure	2.5 (2–4)	0.50	4 (2–4)	0.78	Yes	Ambiguous	4 (2–4)	0.93	4 (2–4)	0.89	No	Retained		Retained	No
Deviating course in the use of medical device	3 (2–4)	0.90	3 (2–4)	0.70	No	Retained	4 (2–4)	0.96	3 (1–4)	0.71	Yes	Retained		Retained	No
Mistake, complaint and incident	4 (4–4)	1.00	4 (2–4)	0.90	Yes	Retained	4 (3–4)	1.00	4 (3–4)	1.00	Yes	Retained		Retained	No
Other	4 (2–4)	0.88	3.5 (2–4)	0.88	Yes	Retained	4 (2–4)	0.93	3 (1–4)	0.79	No	Retained		Retained	No
Transfusion												Added		Retained	No
Endocrine impairment													Added	Retained	No
Medication module															
Adverse drug event/Adverse drug reaction	4 (3–4)	1.00	3 (2–4)	0.71	Yes	Retained	4 (3–4)	1.00	4 (2–4)	0.87	No	Retained		Retained	No
Drug that requires follow–up	4 (2–4)	0.76	2 (1–4)	0.59	No	Ambiguous	4 (1–4)	0.69	4 (2–4)	0.88	No	Removed			
Drug management	3 (2–4)	0.56	3.5 (1–4)	0.75	Yes	Ambiguous	4 (2–4)	0.97	4 (2–4)	0.87	No	Retained		Retained	No
Continuity and transition module															
Unplanned change in care–providing unit, admission and outpatient visit	4 (3–4)	1.00	3 (2–4)	0.67	No	Retained	4 (1–4)	0.85	4 (1–4)	0.82	No	Retained		Retained	No
Unplanned contact with physician and registered nurse	3 (1–4)	0.75	3 (2–4)	0.87		Removed									
Insufficient planning, coordination, communication and information	4 (4–4)	1.00	3.5 (3–4)	1.00	Yes	Retained	4 (2–4)	0.83	4 (2–4)	0.93	No	Retained		Retained	No
Patient treated off–site						Added	4 (1–4)	0.97	4 (2–4)	0.90	No	Removed			

The original version of this article has been updated.

**Error in figure/table**

There was a mistake in table 3 as published. During table assembly, the min–max values were shifted between the three adjacent subcolumns for usefulness of triggers, comprehensibility of trigger definitions, and comprehensibility of decision support. This resulted in multiple incorrect min–max values in the published table. In addition, the word “Ambiguous” was misspelled as “Ambigious” in Tables 2 and 3. The published medians, consensus values, and trigger status remain unchanged. No individual min–max values from Tables 2 and 3 are reported in the Results or Discussion text. The corrections in Tables 2 and 3 do not affect the results, analyses, interpretation, or conclusions of the article. The corrected table 3 appears below.

TABLE 3 Result of the reviewers' survey.

**Table 3:** Result of the reviewers' survey

Modules and triggers	Relevance of triggers		Usefulness of triggers		Comprehensibility of trigger definitions		Comprehensibility of decision support		Result of reviewers’ survey	Changed trigger names or definitions
	Median (min–max)	Consensus	Median (min–max)	Consensus	Median (min–max)	Consensus	Median (min–max)	Consensus		
General Module										
Deterioration in vital functions	4 (3–4)	1.00	3 (3–4)	1.00	4 (3–4)	1.00	4 (3–4)	1.00	Retained	No
Neurological impairment	4 (4–4)	1.00	4 (3–4)	1.00	4 (3–4)	1.00	4 (4–4)	1.00	Retained	No
Blood vessel, skin or tissue impairment	4 (3–4)	1.00	4 (3–4)	1.00	4 (2–4)	0.75	4 (3–4)	1.00	Retained	Yes
Renal impairment	3.5 (3–4)	1.00	3 (2–4)	0.88	4 (3–4)	1.00	4 (3–4)	1.00	Retained	No
Thrombosis or embolus	4 (3–4)	1.00	4 (2–4)	0.75	4 (4–4)	1.00	4 (4–4)	1.00	Retained	Yes
Healthcare–associated infection	4 (2–4)	0.88	4 (2–4)	0.88	4 (3–4)	1.00	4 (2–4)	0.88	Retained	Yes
Gastrointestinal impairment	4 (4–4)	1.00	4 (3–4)	1.00	4 (3–4)	1.00	3 (3–4)	1.00	Retained	No
Impairment of oral health	4 (4–4)	1.00	4 (4–4)	1.00	4 (4–4)	1.00	4 (3–4)	1.00	Retained	No
Distended urinary bladder	3.5 (2–4)	0.88	3 (2–4)	0.88	4 (3–4)	1.00	3.5 (3–4)	1.00	Retained	No
Weight loss	4 (3–4)	1.00	4 (3–4)	1.00	4 (2–4)	0.88	3.5 (3–4)	1.00	Retained	No
Fall	3 (2–4)	0.63	3 (2–4)	0.75	4 (3–4)	1.00	4 (3–4)	1.00	Ambiguous	No
Pain	4 (4–4)	1.00	4 (3–4)	1.00	4 (4–4)	1.00	4 (3–4)	1.00	Retained	No
Psychological impairment	4 (2–4)	0.88	4 (2–4)	0.88	4 (3–4)	1.00	4 (3–4)	1.00	Retained	No
Unplanned invasive procedure or deviating course in invasive procedure	4 (3–4)	1.00	3.5 (2–4)	0.88	4 (2–4)	0.88	4 (3–4)	1.00	Retained	No
Deviating course in the use of medical device	4 (3–4)	1.00	3.5 (3–4)	1.00	4 (2–4)	0.88	4 (3–4)	1.00	Retained	No
Mistake, complaint and incident	4 (4–4)	1.00	4 (3–4)	1.00	4 (4–4)	1.00	4 (3–4)	1.00	Retained	No
Transfusion	4 (3–4)	1.00	4 (2–4)	0.63	4 (4–4)	1.00	4 (3–4)	1.00	Retained	Yes
Endocrine impairment	4 (3–4)	1.00	3.5 (3–4)	1.00	4 (3–4)	1.00	4 (3–4)	1.00	Retained	No
Other	4 (2–4)	0.88	4 (2–4)	0.63	4 (3–4)	1.00	4 (3–4)	1.00	Retained	Yes
Medication module										
Adverse drug event/Adverse drug reaction	4 (4–4)	1.00	4 (2–4)	0.88	4 (4–4)	1.00	4 (3–4)	1.00	Retained	No
Deviating course in drug management	4 (4–4)	1.00	4 (3–4)	1.00	4 (2–4)	0.88	4 (3–4)	1.00	Retained	No
Continuity and transition module										
Unplanned change in care–providing unit	3.5 (2–4)	0.88	2.5 (2–4)	0.50	4 (2–4)	0.88	4 (2–4)	0.88	Retained	Yes
Insufficient planning, coordination, communication and information	4 (2–4)	0.88	3.5 (2–4)	0.88	4 (1–4)	0.88	4 (3–4)	1.00	Retained	Yes

The original version of this article has been updated.

